# Illumina identification of RsrA, a conserved C2H2 transcription factor coordinating the NapA mediated oxidative stress signaling pathway in *Aspergillus*

**DOI:** 10.1186/1471-2164-15-1011

**Published:** 2014-11-22

**Authors:** Jin Woo Bok, Philipp Wiemann, Graeme S Garvey, Fang Yun Lim, Brian Haas, Jennifer Wortman, Nancy P Keller

**Affiliations:** Department of Medical Microbiology and Immunology, University of Wisconsin-Madison, Madison, WI USA; Monsanto Vegetable Seeds, 37437 State Highway 16, Woodland, CA 95695 USA; Genome Sequencing and Analysis Program, Broad Institute of MIT and Harvard, 7 Cambridge Center, Cambridge, MA 02142 USA

**Keywords:** *Aspergillus*, Next generation sequencing, Mutagenesis, Stress response, AP-1, CCAAT

## Abstract

**Background:**

Chemical mutagenesis screens are useful to identify mutants involved in biological processes of interest. Identifying the mutation from such screens, however, often fails when using methodologies involving transformation of the mutant to wild type phenotype with DNA libraries.

**Results:**

Here we analyzed Illumina sequence of a chemically derived mutant of *Aspergillus nidulans* and identified a gene encoding a C2H2 transcription factor termed RsrA for *r*egulator of *s*tress *r*esponse. RsrA is conserved in filamentous fungal genomes, and upon deleting the gene in three *Aspergillus* species (*A. nidulans, A. flavus* and *A. fumigatus*), we found two conserved phenotypes: enhanced resistance to oxidative stress and reduction in sporulation processes. For all species, *rsrA* deletion mutants were more resistant to hydrogen peroxide treatment. In depth examination of this latter characteristic in *A. nidulans* showed that upon exposure to hydrogen peroxide, RsrA loss resulted in global up-regulation of several components of the oxidative stress metabolome including the expression of *napA* and *atfA,* the two bZIP transcription factors mediating resistance to reactive oxygen species (ROS) as well as NapA targets in thioredoxin and glutathione systems. Coupling transcriptional data with examination of *ΔrsrAΔatfA* and *ΔrsrAΔnapA* double mutants indicate that RsrA primarily operates through NapA-mediated stress response pathways. A model of RsrA regulation of ROS response in *Aspergillus* is presented.

**Conclusion:**

RsrA, found in a highly syntenic region in *Aspergillus* genomes, coordinates a NapA mediated oxidative response in *Aspergillus* fungi.

**Electronic supplementary material:**

The online version of this article (doi:10.1186/1471-2164-15-1011) contains supplementary material, which is available to authorized users.

## Background

Living organisms including fungi sense and respond to various environmental stresses. Responses to these stresses can be associated with morphological and chemical differentiation, including sporulation and secondary metabolite production in filamentous fungi [1]. Several studies have correlated the synthesis of specific metabolites and spore development with oxidative stress [2–7]. One system known to coordinate sporulation and development and, more recently oxidative stress, is the heterotrimeric transcriptional regulator known as the Velvet Complex [8, 9].

The best-described members of the Velvet Complex are the methyltransferase LaeA (reviewed in [10]) and the scaffold protein VeA (reviewed in [11]). As both proteins regulate a substantial proportion of the fungal genome [12–14], efforts have been focused on characterizing LaeA and VeA signaling pathways with the goal of thoroughly understanding regulation of either secondary metabolism or differentiation processes in fungi. Consistent with the broad regulation of the transcriptome by the Velvet Complex, LaeA and VeA regulate other aspects of fungal development including the oxidative stress response [5, 15–18]. Efforts in understanding how LaeA and VeA regulate so many processes in fungal development have centered on identifying mutations that are able to restore a phenotype of interest in these deletion mutants (*ΔlaeA* and *ΔveA*). Restoration of sterigmatocystin, a highly produced secondary metabolite in *A. nidulans* regulated by both LaeA and VeA, is often used for screening purposes [19, 20].

Mutations arising from chemical mutagenesis screens, in particular, are both costly and time-consuming to identify through standard DNA library complementation. Recently next generation sequencing methods have been successfully applied to identify mutant genes in a number of major model organisms [21, 22] and advances in these technologies have significantly reduced cost, while making this approach accessible for other genetically tractable systems, including fungal organisms such as *Sordaria macrospora* [23, 24]. Here we describe our identification of a C2H2 transcription factor, termed RsrA, using Illumina technology to interrogate a mutant arising from a 4-nitroquinoline-1-oxide (4-NQO) screen of a *ΔlaeA* mutant.

Although loss of *rsrA* corroborated the original mutation (e.g. restoration of sterigmatocystin synthesis in the *ΔlaeA* background), the primary impact of *rsrA* loss was on resistance to oxidative stress and reduced reproductive development. The *rsrA* allele is located in a syntenic region in the genomes of *Aspergillus* spp. and its function is conserved in *A. nidulans*, *A. flavus* and *A. fumigatus.* In depth exploration of the oxidative stress metabolome in *A. nidulans* supports a model where RsrA governs fungal response to reactive oxygen species (ROS) through the conserved bZIP transcription factor NapA and its downstream targets.

## Results

### Ilumina sequence analysis leads to identification of *A. nidulans rsrA*

Mutagenesis of a *ΔlaeAΔstcEveA1* strain, RJW160.15, led to identification of one mutant, MGG1.124, in which norsolorinic acid was restored to greater than wild-type levels (Additional file 1: Figure S1). Norsolorinic acid (NOR), which accumulates when *stcE* is deleted, is a visible precursor to sterigmatocystin and used as a convenient screen for identifying mutations affecting sterigmatocystin biosynthesis [25]. Five backcrosses to MGG1.124 were made in efforts to minimize SNPs. Two progenies (RJW207.1 and RJW207.3) carrying the same secondary metabolite phenotype as the original mutant (e.g. restored NOR production in a *ΔlaeAΔstcEveA1* background) were sequenced using Illumina technology. Based on SNP analysis, we identified 11 genes with coding region polymorphisms associated with the MGG1.124 mutant. Further culling through two more sexual crosses generating RJW207A, RJW207B, and RJW207C coupled with PCR-based SNP detection enabled us to identify one gene, *AN0273*, encoding a putative C2H2 zinc finger transcription factor as the primary MGG1.124 lesion. Sequence analysis of *AN0273* in MGG1.124 showed it harbored a C -> T transition at codon 163 leading to an early stop codon in the predicted ORF.

**Figure 1:**
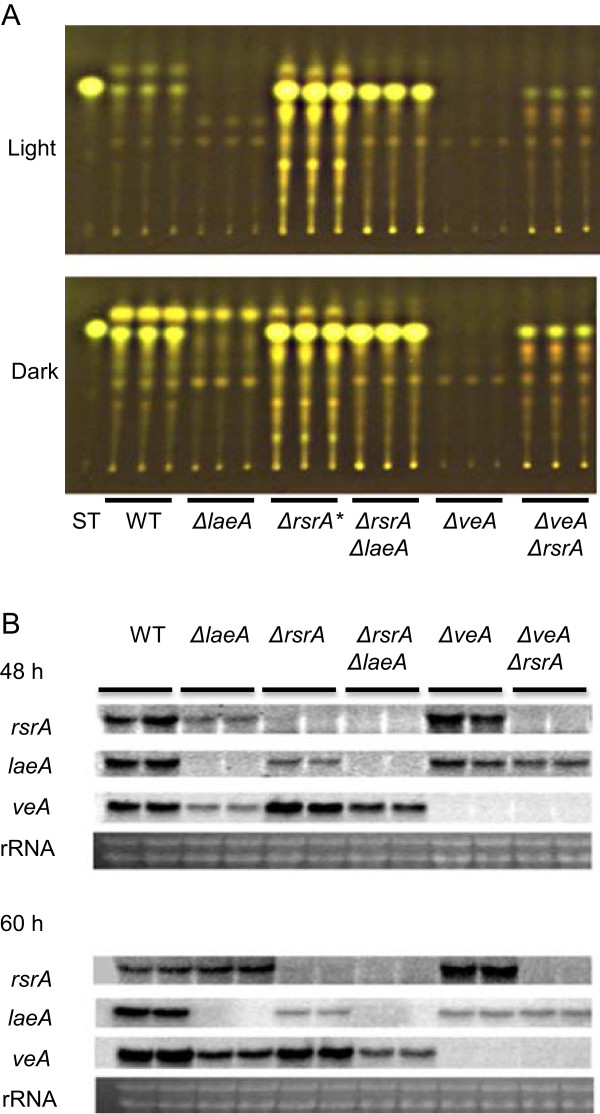
**Sterigmatocystin production and expression analysis of** ***A. nidulans ΔrsrA*** **. (A)** Thin Layer Chromatography analysis of chloroform extracts for metabolite production by the wild type (WT), *ΔlaeA*, *ΔrsrA*, *ΔrsrAΔlaeA*, *ΔveA*, *and ΔveAΔrsrA*, (RDIT9.32, RJW41A, RJW263.2 and RJW273.17, RJW112.2, and RJW113.4, respectively) strains grown on solid glucose minimal media (GMM) under light and dark at 37°C for 5 days in triplicate. ST, sterigmatocystin standard. *: 2.5 μL loading out of 100 μL sample. Others were loaded with 5 μL. **(B)** Gene expression analysis of *A. nidulans* strains, wild type (WT), *ΔlaeA*, *ΔrsrA*, *ΔrsrAΔlaeA*, *ΔveA*, *and ΔveAΔrsrA* grown on liquid GMM at 37°C, 225 rpm for 48 and 60 h in duplicate. Ethidium bromide-stained rRNA is indicated for loading.

The wild type *AN0273* ORF was deleted as determined by Southern analysis and examined in both *veA* and *veA1* backgrounds (wild type *stcE*) with and without an intact *laeA* gene (Additional file 2: Figure S2A). As shown in Figure 1A, extracts from these near isogenic strains revealed that loss of *AN0273* in the *ΔlaeA* background restored sterigmatocystin synthesis, thus supporting the original mutant phenotype. The *ΔAN0273* strain produced considerably more sterigmatocystin than wild type. We also found that *AN0273* loss could partially restore sterigmatocystin in *ΔveA* (Figure 1A) and that, dependent on the time point examined, *AN0273* was mis-regulated in both *ΔveA* and *ΔlaeA* backgrounds (Figure 1B).

Despite the impact on sterigmatocystin and its precursors, other secondary metabolites were not greatly impacted in these growth conditions nor did deletion of this gene have a significant effect on secondary metabolism in *A. fumigatus* and *A. flavus* (data not shown). Hence, *AN0273* was named *rsrA* (*r*egulator of *s*tress *r*esponse with mutated allele named *rsrA163*) for its association with the stress response in Aspergilli as detailed below.

### RsrA is required for both meiotic and mitotic spore development

**Figure 2:**
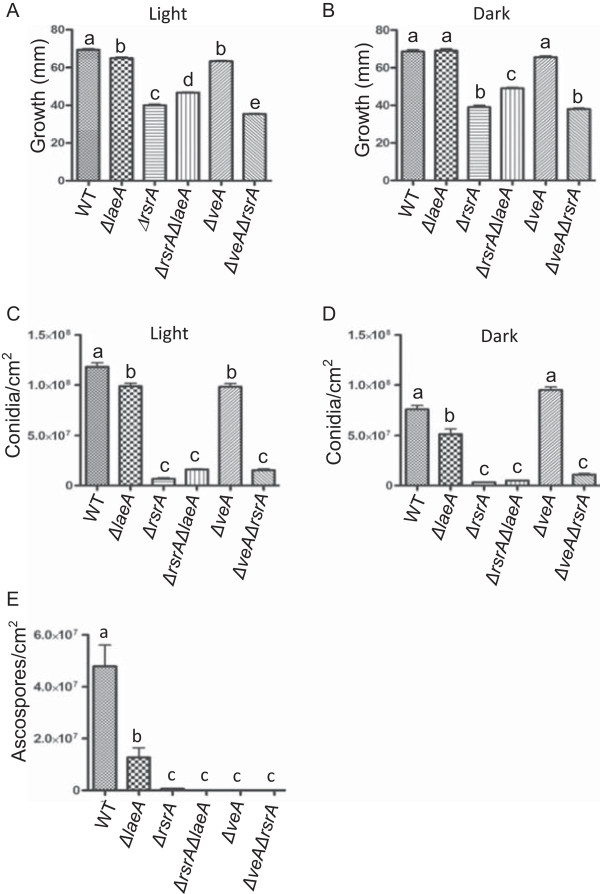
**Average radial growth, and sexual and asexual spore production of** ***A. nidulans*** **strains, WT,** ***ΔlaeA*** **,** ***ΔrsrA*** **,** ***ΔrsrAΔlaeA*** **,** ***ΔveA*** **, and** ***ΔveAΔrsrA*** **(RDIT9.32, RJW41A, RJW263.2 and RJW273.17, RJW112.2, and RJW113.4 respectively).** Average radial growth on solid GMM under light **(A)** and dark **(B)** at 37°C for 5 days in triplicate, respectively. Asexual spore production of each strain grown at 37°C for 5 days in the light **(C)** and dark **(D)**, respectively. Sexual spore production after 5 days in the dark **(E)**. Error bars indicate standard deviations for triplicates of each strain calculated by analysis of variance (ANOVA). Different letters indicate statistically differences (P < 0.05) according to Tukey’s multiple comparison test.

Sterigmatocystin production is coupled with both asexual and sexual sporulation in *A. nidulans* and therefore we examined both mitotic and meiotic spore production as well as general growth in the *ΔrsrA* mutant. The *ΔrsrA* strain exhibited a sick growth phenotype, with reduced radial growth; this reduction in radial growth was apparent in all double mutants as well (Additional file 3: Figure S3 and Additional file 4: Figure S4). An examination of conidia and ascospore production showed that *rsrA* loss nearly eliminated production of both spores, regardless of genetic background (Figure 2). Complementation of *ΔrsrA* with its wild-type allele largely restored phenotypes to that of wild type levels for growth and sporulation patterns; however sterigmatocystin production was only partially restored (Additional file 3: Figure S3).

### RsrA is a conserved protein affecting development in Aspergilli including spore (*A. fumigatus, A. flavus*) and sclerotia (*A. flavus*) production

Putative RsrA orthologs are located in a highly syntenic region of multiple Aspergilli genomes (Additional file 5: Figure S5). We thus wondered if the protein might play any similar role in other Aspergilli and therefore deleted the putative ortholog in two *A. fumigatus* strains and one *A. flavus* strain (Southern blots shown in Additional file 2: Figure S2B, C, and D). A visual examination of the mutants showed a distinct phenotype in the *A. flavus* mutant and in the CEA17 KU80Δ but not the Af293 *A. fumigatus* background (Additional file 6: Figure S6).

**Figure 3:**
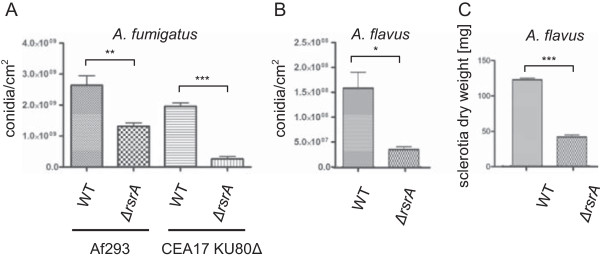
**Reproductive development in** ***A. flavus*** **and** ***A. fumigatus.*** **(A)** Asexual spore production of the respective *A. fumigatus* strain. Error bars indicate standard deviations for triplicates of each strain. Asterisks indicate significance as calculated by unpaired *t* test: ** = p-value <0.01; *** = p-value <0.0001. **(B)** Asexual spore production of the indicated *A. flavus* strains. Error bars indicate standard deviations for triplicates of each strain. Asterisk indicates significance as calculated by unpaired *t* test: * = p-value <0.05. **(C)** Sclerotia production in *A. flavus.* Solid GMM with 2% sorbitol medium for sclerotia production was inoculated with 10^4^ spores and incubated for 7 days in the dark. Error bars indicate standard deviations for triplicates of each strain calculated by ANOVA. Asterisk indicates significance as calculated by unpaired *t* test: *** = p-value <0.0001.

As one major impact of *rsrA* loss on *A. nidulans* was on reproduction development, we assessed asexual sporulation in the *A. fumigatus* and *A. flavus* mutants and sclerotia production in the latter species. All three *rsrA* deletion strains were examined for spore production. Figure 3A and B illustrate that similar to *A. nidulans*, both *A. fumigatus* and *A. flavus ΔrsrA* strains displayed reduced conidia production. Additionally, *rsrA* loss greatly reduced sclerotia production in *A. flavus* (Figure 3C). Sclerotia are analogous developmental structures to cleistothecia and can house ascospores when opposite mating types of *A. flavus* are paired [26]. Sexual development was not examined in the *A. fumigatus* strain due to the length of time needed to assess this process in this species [27].

### A conserved role for RsrA in sensitivity to hydrogen peroxide

**Figure 4:**
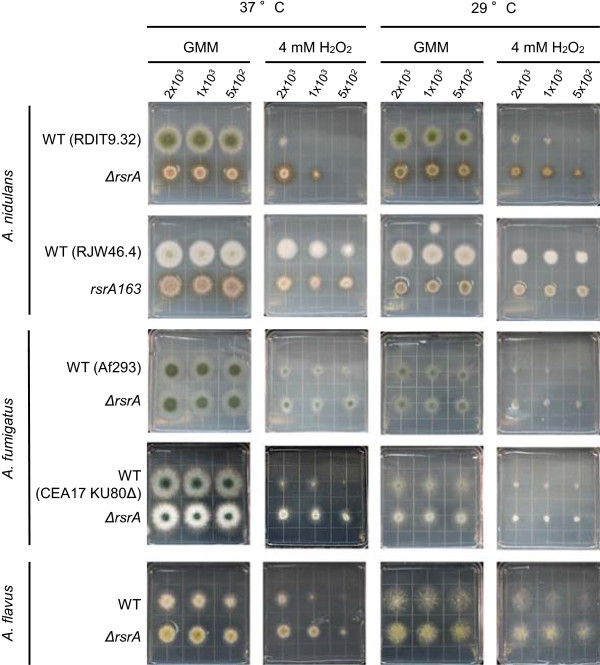
**Growth phenotypes in response to H**_**2**_ **O**_**2**_ **of** ***A. nidulans*** **,** ***A. fumigatus*** **and** ***A. flavus*** **wild-type strains, respective** ***rsrA*** **deletants and the** ***A. nidulans*** **mutant RJW207.B (** ***rsrA163*** **).** Serial dilutions of the indicated strains were point-inoculated on GMM with or without 4 mM H_2_O_2_ and incubated in the dark at 29°C and 37°C, respectively.

As the *rsrA* gene was induced by camptothecin in a study assessing DNA damage in *A. nidulans* [28], we thought RsrA might be involved in a stress response in *A. nidulans* and possibly other Aspergilli. Therefore, *ΔrsrA* mutants and their wild type controls were assessed for response to a variety of challenges including exposure to hydrogen peroxide (H_2_O_2_), *tert*-butyl hydroperoxide (*t*BOOH), camptothecin, alkaline pH, sodium chloride, sorbitol and congo red (Figure 4, Additional file 7: Figure S7).

**Figure 5:**
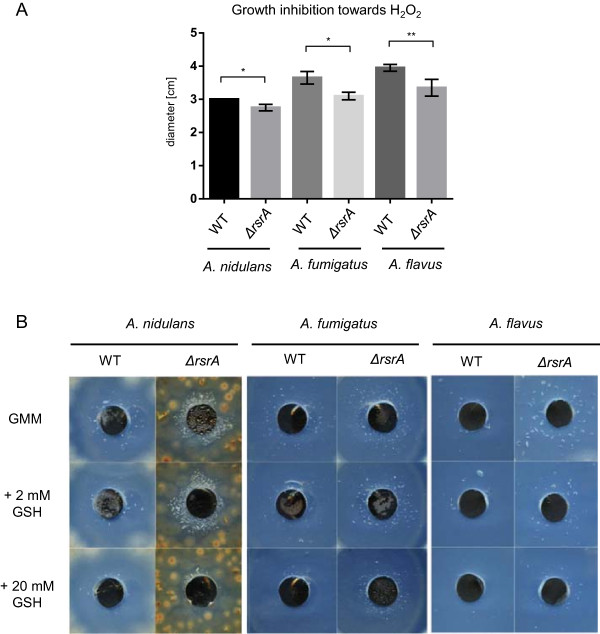
**Hydrogen peroxide diffusion assay and catalase activity assay. (A)** Diameter of growth inhibition of the indicated strains after 24 h at 37°C (*A. nidulans* and *A. fumigatus* Af293) and 29°C (*A. flavus*). Asterisks indicate significance as calculated by unpaired *t* test: * = p-value <0.05; ** = p-value <0.01. **(B)** Air bubble formation indicative of catalase activity in the growth inhibition zone after 48 h of the indicated strains.

A conserved response was observed to hydrogen peroxide challenge where all four *ΔrsrA* mutants – although not the 6^th^ backcross progeny mutant RJW207B (*rsr163*) possibly due to containing *veA1* and *ΔlaeA* alleles - grew better than wild type (Figure 4). Supporting the results from the growth assay on hydrogen peroxide, a hydrogen peroxide diffusion assay showed a reduced zone of growth inhibition in the *ΔrsrA* mutants compared to the respective wild type strains of *A. nidulans*, *A. fumigatus* and *A. flavus* (Figure 5A). Additionally, in the zones of growth inhibition an increased formation of air bubbles was observed in the *ΔrsrA* mutants compared to the respective wild types of all the Aspergilli (Figure 5B). Fungi produce catalases in order to detoxify hydrogen peroxide into molecular oxygen and water [29], thereby forming oxygen air bubbles in the solidified agar as shown in Figure 5B. Addition of increasing concentrations of the reducing agent glutathione (GSH) decreased air bubble formation in all strains, but the formation was still more apparent in the *ΔrsrA* strains compared to their respective wild types.

### RsrA regulates critical stress transcriptional pathways in *A. nidulans*

Considering that the conserved phenotypes of the *rsrA* mutants in the three *Aspergillus* spp. examined center around hydrogen peroxide resistance and development (which is known to involve ROS species as signaling molecules [7, 30]), we set out to examine a possible role for RsrA in ROS biology. Therefore, we assessed expression of genes associated with resistance to ROS in *A. nidulans.* This included transcriptional regulators *napA* and *atfA* (conserved bZIP oxidative response elements in eukaryotes, [24, 31–35]) and members of the CCAAT-binding factor AnCF (*hapB, hapE* and *hapC*, [32]) as well as components of the thioredoxin system (*trxA, trxR* and *prxA*, [36]), the glutathione system (*glrA, gstA* and *gpxA*, [37, 38]), and two representative detoxification enzymes *catB* (mycelial catalase, [39]) and *sodA* (superoxide dismutase, [40]).

**Figure 6:**
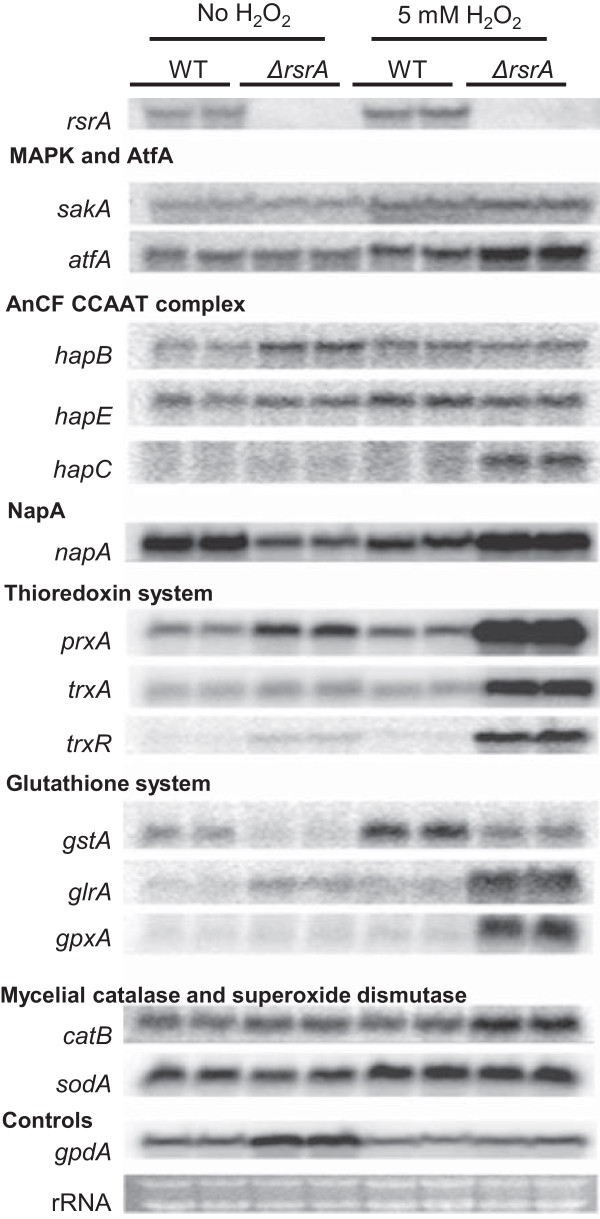
**Gene expression analysis of** ***A. nidulans*** **strains, WT (RDIT9.32) and** ***ΔrsrA*** **(RJW263.2) grown on 20 mL liquid GMM at 37°C** **, 225 rpm for 18 h and additional 30 min culture after adding 5 mM H**_**2**_ **O**_**2**_ **in duplicate.** Ethidium bromide-stained rRNA and *gpdA* expression are indicated for loading.

As shown in Figure 6, many of these genes were highly up-regulated when *ΔrsrA* was exposed to hydrogen peroxide. *napA* was several fold up-regulated as were several NapA target genes in thioredoxin and gluthathione metabolism (with the exception of *gstA* which was downregulated) but not NapA targets *catB* and *sodA. atfA* was also up-regulated although transcripts of *sakA* encoding the AtfA MAPK interacting partner appeared not to be affected by RsrA loss [33]. Of the AnCF subunits, only *hapC* was upregulated in *ΔrsrA.*

### RsrA coordinates ROS response through NapA

**Figure 7:**
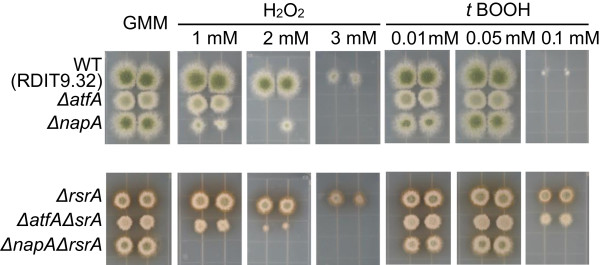
**Growth phenotypes in response to H**_**2**_ **O**_**2**_ **of** ***A. nidulans*** **wild-type strain and indicated mutants.** Strains were point-inoculated on GMM with or without indicated concentrations of H_2_O_2_ and *t*BOOH and incubated in the dark at 37°C for 48 h.

The transcriptional data suggested a possible role for NapA or AtfA in mediation of the RsrA ROS affects. To elucidate such a possibility, we next made double mutants of *ΔnapA* and *ΔatfA* with *ΔrsrA* and assessed the double mutants to their respective controls for growth on hydrogen peroxide and *t*BOOH (Figure 7). The double mutant *ΔatfAΔrsrA* but not *ΔnapAΔrsrA* demonstrated an enhanced resistance to both oxidative stressors, thus indicative of NapA as the primary conduit of RsrA integration to ROS regulation.

## Discussion

Whole-genome sequencing is now a feasible method to identify mutations associated with a desired phenotype [21, 22, 24, 41, 42]. In *Aspergillus* spp. and other fungi, single gene mutations have been typically identified through meiotic mapping and/or complementation with DNA libraries [43]. This is an arduous procedure requiring a significant amount of time and effort that does not always meet with success. Given the relatively small genome size of model fungi coupled with the decreasing costs of DNA sequencing, we thought it feasible to utilize Illumina whole genome sequence to identify an *A. nidulans* mutation, obviating the need for prior characterization of informative markers. We utilized the sequenced strain of this species, employing a chemical mutagen to generate the mutation of interest, which we then backcrossed to reduce the number of SNPs to investigate. This approach was successful, identifying a small and tractable number of candidate mutations to test, revealing RsrA as a conserved protein regulating the oxidative stress response and reproductive development in Aspergilli.

RsrA encodes a putative C2H2 zinc finger transcription factor. C2H2 proteins are conserved in eukaryotes, falling into an estimated 37 gene families [44]. *A. nidulans* contains 60 such proteins in its genome [45]. The two primary phenotypes of *rsrA* loss, decrease in sporulation and enhanced resistance to oxidative stress, were conserved in the three species examined. Deletions in all species resulted in significant decrease in asexual spores as well as sexual structures/spores in *A. nidulans* and *A. flavus* (Figures 3 and 4). Considering the many studies linking sporulation and ROS [4, 7, 46, 47], these aberrancies in development may be genetically linked with the altered oxidative stress response in the *ΔrsrA* background.

The oxidative stress response in fungi has been the topic of many studies due to the importance of this pathway in several aspects of fungal biology. Maintaining a mechanism to detoxify ROS is critical for all forms of aerobic life and ROS are mediators of cell signaling processes governing differentiation processes. Hence studies have been directed at understanding the processes regulating this response in fungi including *Aspergillus.* Here we sought to place RsrA in the context of known ROS pathways. Transcriptional regulation of the oxidative response pathway is mediated by two well-known bZIP proteins, NapA (called Yap1 in other filamentous fungi) and AtfA, and the CCAAT binding complex AnCF. RsrA acts as a repressor of both bZIP genes and one subunit of the AnCF complex during hydrogen peroxide treatment (Figure 6). However, considering that *hapB* and *hapE* transcripts were not affected in *ΔrsrA* and that HapC would be oxidized by H_2_O_2_ and thus inactive, AnCF is unlikely a mediator of RsrA regulation of the oxidative stress response.

**Figure 8:**
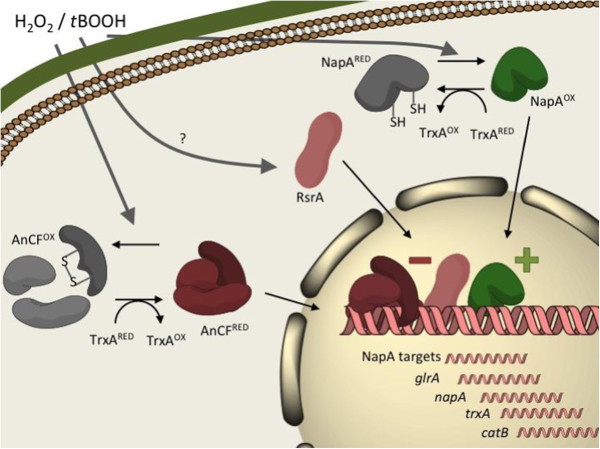
**Model integrating RsrA into the global ROS-responsive network in** ***A. nidulans.*** Upon hydrogen peroxide exposure, the bZIP transcription factor NapA gets directly oxidized and thereby activates gene expression of enzymes and components (TrxA, CatB) involved in balancing the redox status of the cells. RsrA acts as a repressor of certain NapA-activated target genes, perhaps in balance with the AnCF complex. How RsrA is activated itself remains unclear.

Of the two bZIP proteins, our accumulative data suggests NapA and not AtfA to be the prime conduit of RsrA signaling (illustrated as a model in Figure 8). NapA is directly activated by oxidation through ROS, allowing it to enter the nucleus and exert its role as a positive transcription factor. Previous studies in *Aspergillus* spp. have supported a role for NapA in positively regulating both non-enzymatic (e.g. glutathione and thioredoxin) and enzymatic pathways (e.g. catalases and superoxide dismutases) important in protection against ROS [30–32]. Although *catB* and *sodA* were not greatly – if at all – affected by *rsrA* loss, all members of the thioredoxin and gluthathione metabolism, excluding *gstA*, were highly upregulated during H_2_O_2_ treatment of *ΔrsrA.* GlrA (glutathione reductase) and GpxA (glutathione peroxidase) are key components of the glutathione/glutathione disulfide (GSS/GSSH) cycle in fungi [38, 48] whereas GstA (glutathione transferase) transfers GSH to xenobiotic substrates for detoxification [37, 49]. Thus the differential response of these three genes may implicate RsrA in balancing the GSS/GSSH ratios, at least during ROS stress. Assessment of ROS sensitivity of the double mutants support a major role for NapA in regulating ROS detoxification mechanism (Figure 7) and clearly place RsrA in epistatic relationship with NapA. By induction of genes involved in ROS detoxification, NapA is the activator of an intrinsic negative feedback loop counteracting oxidative stress and hence its own deactivation by reduction [32].

Our collective data suggest that deletion of *rsrA* results in increased tolerance towards hydrogen peroxide stress, possibly due to increased expression levels of genes involved in GSH and thioredoxin production (Figure 6) as well as increased catalase activity (Figure 5). This increased activation is most likely through NapA (Figures 7 and 8). As an increased arsenal of reducing agents (GSH, thioredoxin and catalases) would result in inactivation of NapA in the wild type [32], RsrA loss somehow leads to an active form of NapA that results in increased target gene expression even when ROS stress is counteracted. Therefore we suggest that RsrA could be a negative transcriptional element in the global regulatory network of oxidative stress response, perhaps in balance with the AnCF complex (Figure 8).

## Conclusion

In summary, we have successfully identified the putative C2H2 protein RsrA using state of the art whole genome sequencing methodologies. RsrA is negative regulator of the oxidative response cascade acting primarily through inhibition of NapA and NapA target activities.

## Methods

### Fungal culture

Strains used or created in this study are listed in Table 1. All strains unless otherwise noted were grown on glucose minimal media (GMM) [50], with additional supplements for auxotrophic strains (pyrodoxin, riboflavin, tryptophan, uridine or uracil as needed). All strains are maintained as glycerol stocks at -80°C.

**Table 1:** **Genotypes and sources of strains used or created in this study**

Strain	Genotype	Reference
*A. nidulans*		
MGG1.124	*ΔlaeA::metG, ΔstcE::argB, trpC801, wA3, rsrA163, veA1*	this study
RDIT2.1	*metG1*	[43]
RDIT9.32	WT	[51]
RJH0126	*ΔstcE::argB; argB2; trpC801; biA1; wA3; veA*1	this study
RJW2	*pyrG89, ΔstcE::argB, wA3, veA1*	this study
RJW41A	*ΔlaeA::metG*	[8]
RJW46.4	*ΔlaeA::metG, veA1*	[43]
RJW112.2	*ΔveA::argB*	this study
RJW113.4	*ΔveA::argB, pyrG89*	[52]
RJW160.17	*trpC801, ΔstcE:;argB, ΔlaeA::metG, wA3, veA1,*	this study
RJW207.1, 3	*ΔstcE::argB, ΔstcE::argB, wA3, veA1, rsr163* (5^th^ cross progeny)	this study
RJW207A	*ΔstcE::argB, ΔstcE::argB, wA3, veA1, rsr163* (6^th^ cross progeny)	this study
RJW207B	*ΔlaeA::metG, veA1, rsrA163* (6^th^ cross progeny)	this study
RJW207C	*ΔstcE::argB, ΔstcE::argB, wA3, rsr163* (6^th^ cross progeny)	this study
RJW263.2	*ΔrsrA:: A. parasiticus pyrG*	this study
RJW273.17	*ΔrsrA:: A. parasiticus pyrG, ΔlaeA::metG*	this study
RJW277.13	*ΔrsrA:: A. parasiticus pyrG, pyroA4*	this study
RJW279.2	*ΔveA::argB, ΔrsrA:: A. parasiticus pyrG*	this study
RTMH207.13	*pyrG89*	[53]
TNO2A7	*nku70::argB, riboB2, pyrG89, pyroA4, veA1*	[54]
TJW131	*nku70::argB, ΔrsrA::A. parasiticus pyrG, riboB2*, *pyrG89, pyroA4, veA1*	this study
TJW150.3	*ΔrsrA::pyrG*, *rsrA::pyroA*	this study
HZS189	*ΔatfA::riboB, pabaA1, riboB2, veA1*	[55]
TWY7.3	*ΔnapA::pyroA, ∆nkuA::argB, pyrG89, pyroA4*	[30]
RFYL9.1	*ΔnapA::pyroA*	this study
RFYL10.1	*ΔatfA::riboB*	this study
RJW297.2	*ΔrsrA:: A. parasiticus pyrG, ΔatfA::riboB*	this study
RJW298.10	*ΔrsrA:: A. parasiticus pyrG, ΔnapA::pyroA*	this study
*A. flavus*		
NRRL3357	WT	[56]
NRRL3357.5	*pyrG-*	[56]
TJW146.1	*ΔrsrA:: A. parasiticus pyrG*	this study
*A. fumigatus*		
CEA17 KU80	*ΔpyrG, pyrG1*, *ΔakuB::pyrG, pyrG1*	[57]
CEA17 KU80 pyrG+	*ΔpyrG1*, *ΔakuB:pyrG*	[57]
TPHW1.4	*pyrG1*, *ΔakuB::pyrG*, *ΔrsrA::pyrG*	this study
Af293	WT	[58]
Af293.1	*pyrG1*	[58]
TPHW26.11	*pyrG1*, *ΔrsrA::pyrG*	this study
TJW55.1	*pyrG1, A. parasiticus pyrG*	[59]

### 4-NQO mutagenesis and backcrossing

500 μL of a conidial suspension (4 × 10^6^ spores/mL) of RJW160.17 was treated with the chemical mutagen, 4-NQO at 20 μM for 30 min at 37°C following described protocols [60]. The spore suspension was centrifuged at 5000 g for 3 min and resuspended in 5% Sodium thiosulfate and incubated for 10 min at room temperature. The spores were then centrifuged down as above and resuspended in 1 mL 0.01% Tween-80, this was repeated two more times. This treatment resulted in 70% conidial killing. The mutagenized conidia were resuspended in Tween water to 1×10^3^ spores /mL. 100 μL of this suspension was then spread plated to 1% Oatmeal + tryptophan agar plates [25]. Oatmeal agar was used to allow for the visualization of NOR, the orange precursor to sterigmatocystin.

Approximately 100,000 conidia were screened on Oatmeal + tryptophan agar for restoration of norsolorinic acid production. NOR producers were grid plated onto fresh Oatmeal + tryptophan plates along with a positive control of RJH0126 (NOR +) and negative control of RJW160.15 (NOR -). The development of norsolorinic acid coloring was then monitored for several days. Those mutants that showed NOR coloring over the time course were then re-plated to GMM and incubated in the dark for 5 days. A center core of each mutant was then extracted with chloroform and the presence of NOR examined by TLC. Those that conclusively showed the recovery of NOR on GMM were single spore isolated. We chose one of those mutants, MGG1.124, to cross with RJW2. One trp- progeny producing NOR on oatmeal media was backcrossed again to RJW2 and this procedure continued for 4 more backcrosses. The final progenies, called RJW207.1 and 207.3, were sequenced using Illumina technology.

### Illumina sequencing and SNP detection

We aligned Illumina WGS reads to the *A. nidulans* genome sequence using Burroughs Wheeler Aligner. SNPs were called using two methods: mpileup of Sam tools [61] and UnifiedGenotyper of GATK [62]. The identified candidate SNPs were manually reviewed in the context of the genome-aligned reads, as visualized using GenomeView [63]. The impact of the SNPs on coding gene sequences, such as missense or nonsense mutations, was assessed using a custom PERL script (publically available at http://sourceforge.net/projects/vcfannotator/). To narrow down alleles of candidate SNPs, sixth progenies (RJW207A, RJW207.B and RJW207C) were created by crossing RJW207.1 to RDIT2.1 and the candidate genes’ PCR products were sequenced to obtain AN0273 (*rsrA*) as the mutated gene.

### Creation of rsrA mutants

All of primers used to create the strains below are listed in Additional file 8: Table S1.

### Aspergillus nidulans

The *rsrA* deletion strain (TJW131) was created in strain TNO2A7 by replacing the AN0273 (*rsrA*) open reading frame with *A. parasiticus pyrG* using modified double joint PCR [64] consisting of the following: 1 kb DNA fragment upstream of the *rsrA* start codon (primers 8797F5 and 8797PR5), a 2 kb DNA fragment of *A. parasiticus pyrG* (primers parapyrGF and parapyrGR) via pJW24 [65], and a 1 kb DNA fragment downstream of the *rsrA* stop codon (primers 8797PF3 and 8797R3). 30 μL of Sephadex® G-50 purified third round PCR product was used for fungal transformation. Polyethylene glycol based fungal transformation was done essentially as described by [43]. *rsrA* deletants were confirmed by PCR and Southern blot and one correct transformant, TJW131.1, was used for sexual crosses. TJW131.1 was sexually crossed with RTMH207.13 to obtain the prototroph RJW263.2. The recombinants were confirmed by PCR. Subsequent northern analysis was done using radiolabeled probes for the corresponding transcript (1 kb DNA fragment, primers 0273IF and 0273IR).

A *rsrA* complementation cassette, pJW159.1 was created by inserting a 4.7 kb PCR product using the primer pair HindrsrAcompF/KpnrsrAcompR and cloning the subsequent product into *Hind*III/*Kpn*I sites of pJW53. pJW159.1 was used to transform RJW277.13 to complement a deletion of *rsrA* allele in the *pyroA* locus. The resulting strain was called TJW150 and confirmed by PCR and Southern blotting (Additional file 3: Figure S3B).

The *ΔlaeAΔrsrA* double mutant strain was created by crossing TJW131.1 with RJW135.1 to obtain RJW273 and confirmed by PCR. *ΔveAΔrsrA* double mutant was created by crossing RJW277.13 with RJW113.4 to obtain RJW270.2. HZS189 and TWY7.3 were crossed to generate both the *ΔnapA::pyroA* (RFYL9.1) and *ΔatfA::riboB* (RFYL10.1) mutants. *ΔrsrAΔnapA* (RJW297.2) and *ΔrsrAΔatfA* (RJW298.10) double mutants were obtained by crossing RJW277.13 to HZS189 and TWY7.3, respectively. RJW Progenies were screened for the WT *veA* allele by growth on GMM for 3 days at 37°C in complete darkness as described in Mooney and Yager [66]. Primers used to determine the genotype of the progenies are listed in Additional file 8: Table S1.

### Aspergillus flavus

The putative *A. flavus rsrA* ortholog was determined by blasting the *A. flavus* genome with RsrA. One hit, AFL2G_00759 (BLASTP: 33% identity; e-value =2^-18^), was identified. AFL2G_00759 was deleted by replacing it with *A. parasiticus pyrG* in the *A. flavus* strain 3357.6 using modified double joint PCR. Construction of the *AflrsrA* deletion consisted of the following: a 1 kb DNA fragment upstream of the *AflrsrA* start codon (primers frsrA5F and frsrA5R), a 2 kb DNA fragment of *A. parasiticus pyrG* (primers parapyrGF and parapyrGR) from pJW24 and a 1 kb DNA fragment downstream of the *AflrsrA* stop codon (primers frsrA3F and frsrA3R). 30 μL of Sephadex® G-50 purified purified third round PCR product was used for fungal transformation. Polyethylene glycol based fungal transformation was performed as for *A. nidulans* [43]. The deletion mutants (TJW146 series) were confirmed by PCR and Southern blot.

### Aspergillus fumigatus

Similar to *A. flavus,* the putative *A. fumigatus rsrA* ortholog was determined by blasting the *A. fumigatus* genome with *A. nidulans* RsrA. One hit, Afu1g02870 (BLASTP: 43.2% identity; e-value =3^-79^), was identified. This gene was deleted in two *A. fumigatus* strains, *A. fumigatus* CEA17 KU80Δ *pyrG-* strain [67] and in *A. fumigatus* Af293.1 [58]. The *A. fumigatus* deletion cassettes were constructed using a double-joint fusion PCR approach as described above. Briefly, approximately 1 kb fragments flanking the targeted deletion region were amplified by PCR from *A. fumigatus* strain CEA17 KU80Δ genomic DNA using primer pairs Afu1g02870_KO_5F/_5R and Afu1g02870_KO_3F/_3R. The *A. parasiticus pyrG* marker gene was amplified from the plasmid pJW24 using the primer pair PWpyrGpromF/PWpyrGtermR. The three fragments were subjected to fusion PCR to generate deletion cassettes. Transformation of *A. fumigatus* was performed as previously described [68]. Single integration of the transformation construct was confirmed by Southern analysis using P^32^-labelled probes created by amplification of the respective deletion construct using primer pair Afu1g02870-KO_5F/-3R.

### Nucleic acid analysis

DNA extraction, restriction enzyme digestion, gel electrophoresis, blotting, hybridization, and probe preparation were performed by standard methods [69]. RNA extractions were made from mycelia of cultures where 10^6^ spores/mL were grown in 50 mL liquid GMM at 37°C with shaking at 250 rpm for 48 and 60 h. Total RNA was extracted using Isol-RNA Lysis Reagent (5 Prime) according to the manufacturer's instructions with approximately 30 μg of total RNA for RNA blot analysis. DNA and RNA blots were hybridized with *rsrA, aflrsrA, fumrsrA, veA, laeA, hapC, atfA, napA, prxA, gpxA, trxA, trxR, catB, glrA, sodA, gstA,* and *gpdA* DNA fragments which were generated by PCR using gene-specific primers as shown in Additional file 8: Table S1.

### Sterigmatocystin and norsolorinic acid analysis

Thin layer chromatography (TLC) was used to assess sterigmatocystin and norsolorinic acid production. Those metabolites were extracted from GMM solid medium culture, which was point-inoculated with 10^3^ spores/plate and grown for 5 days at 37°C. One cm cores were punched from the center of point-inoculated plates and homogenized with 3 mL double distilled H_2_O. Three mL of chloroform were added and samples were centrifuged for 10 min. The organic layer was removed and put into a 3 mL glass vial and left to sit and dry in a fume hood overnight. Dried extracts were resuspended with 100 μL chloroform and 5 or 10 μL were loaded onto a non-UV coated TLC plate. Sterigmatocystin or norsolorinic acid was spotted as a standard. The plates were run in chloroform:acetone (8:2) solvent and stained with 15% aluminum chloride in 95% ethanol. TLC plates were viewed under 254 nm UV light.

### Physiological analysis

One thousand conidia from strains were point-inoculated onto solid GMM in four replicates and incubated in continuous dark at 29°C for 7 days and at 37°C for 5 days. Radial growth was assayed by measuring the diameter of point-inoculated colonies after 5 days and 7 days of incubation at 37°C and 29°C, respectively. Per strain, center cores (1 cm diameter) from three independent plates were excised and each core was homogenized in 3 mL H_2_O containing 0.01% Tween-80 before asexual spores were quantified using a hemocytometer. For assessment of sexual development in *A. nidulans*, 10^6^ spores/5 mL in GMM containing 7.5 g/L agar were plated on solidified GMM and incubated at 37°C for 5 days in the dark. Per *A. nidulans* strain, a core (1 cm diameter) from four independent plates were excised and each core was homogenized in 3 mL H_2_O containing 0.01% Tween-80 before sexual spores were quantified using a hemocytometer. For assessment of asexual spore formation in *A. fumigatus and A. flavus*, 10^7^ spores/5 mL GMM containing 3.5 g/L agar were plated on solidified GMM and grown for 5 days at 37°C in the dark. Counting of spores was performed in accordance to the method for *A. nidulans* as described above.

Sclerotia formation was measured for fungal strains following previously described methods [70]. Briefly, 10 mL of 2% sorbitol GMM media with 1.6% agar was overlaid with 3 mL of 2% sorbitol GMM media with 0.75% agar containing 10^3^ spores/plate of each *A. flavus* strain. Cultures were grown at 29°C under complete darkness for 7 days. To visualize sclerotium formation, plates were sprayed with 70% ethanol. The exposed sclerotia were then collected, lyophilized, and weighed (dry weight per plate). Sclerotial weight was determined by using four replicates.

### Stress tests

For stress tests, indicated number of spores of each strain were spotted on solidified GMM containing either indicated concentrations of H_2_O_2_, *t*BOOH, 1 M NaCl, 2 mg/mL Congo red, 50 μM camptothecin, phosphate buffer pH8, or no stressor. The plates were incubated at 29°C and 37°C for 48h or 72 h in the dark, respectively. The hydrogen peroxide diffusion assay was carried out as initially described in Thön et al., 2007 [36]. For gene expression analysis under H_2_O_2_ stress, total RNA was extracted from biologically duplicated freeze-dried mycelia of *A. nidulans* strains grown on 20 mL liquid GMM at 37°C, 225 rpm for 18hr and additional 30min culture after adding 5 mM H_2_O_2_ in the GMM liquid media.

### Statistical analysis

Statistical differences were analyzed using the GraphPad Prism 5 software package (GraphPad Software, Inc, San Diego, CA).

### Ethics statement

We did not use human subjects at all.

## Electronic supplementary material

Additional file 1: Figure S1: Thin Layer Chromatography analysis of chloroform extracts for metabolite production by the wild type (WT), *ΔlaeA* and the 4-NQO generated mutant MGG1.124. Metabolites were extracted from point-inoculated solid cultures grown for 5 days at 37°C in the dark. NOR, Norsolorinic acid. Extracts of MGG1.124 were diluted 1:5 before loading. Extracts from other strains were loaded undiluted. (PPTX 153 KB)

Additional file 2: Figure S2: Southern confirmation of *rsrA* deletion mutant in *A. nidulans, A. flavus* and *A. fumigatus*. **(A)** *A. nidulans AN0273:* Genomic DNA was digested by *Nco*I (all correct). #1 for subsequent experiment. WT: 9.3kb, *ΔrsrA*: 3 and 7kb. **(B)** *A. flavus* A*FL2G_00759* : Genomic DNA was digested by *BglI*I (#1, 5, 6 correct). #1 for subsequent experiment. WT: 6kb and 3.2kb. *ΔrsrA*: 9kb **(C)** *A. fumigatus* CEA17 AFUB_003250: Genomic DNA was digested with *EcoR*I (#1, 4, 6 correct). #4 chosen for subsequent experiments. WT: 3.5 kb; *ΔrsrA*: 2.1, 3.7 and 3.9 kb. **(D)** *A. fumigatus* Af293 Afu1g02870: Genomic DNA was digested with *Hind*III (#2 correct). #2 was chosen for subsequent experiments. WT: 5.5 kb.; Δ*rsrA*: 1.8, 4.5 and 5 kb. (PPTX 467 KB)

Additional file 3: Figure S3: Complementation of *ΔrsrA* mutant with WT *rsrA* in *A. nidulans*. **(A)** Growth phenotype. **(B)** Southern confirmation *ΔrsrA* complementation by restriction enzyme digestion with *Kpn*I and *Hind*III expected to have 4.7 kb WT *rsrA* band in *ΔrsrA* background (3.2 and 2.6 kb bands). **(C)** TLC analysis of WT, *ΔrsrA* and complemented strain grown on solid GMM under dark at 37°C for 5 days in triplicate. ST, sterigmatocystin standard. Compl = complemented. **(D)** Radial growth, **(E)** Asexual spore production; **(F)** Sexual spore production. Radial growth and spore counts were measured after 5 days of incubation. Means ± standard deviations are indicated for triplicates of each strain. Levels not connected by same letter are significantly different (P < 0.05) according to Tukey’s multiple comparison test. (PPTX 2 MB)

Additional file 4: Figure S4: Growth phenotypes of WT, *ΔlaeA*, *ΔrsrA*, *ΔrsrΔlaeA*, *ΔveA*, and *ΔveAΔrsrA* (RDIT9.32, RJW41A, RJW263.2 and RJW273.17, RJW112.2, and RJW113.4, respectively) strains grown on solid GMM under light and dark at 37°C for 5 days. (PPTX 1 MB)

Additional file 5: Figure S5: Synteny of *rsrA* in *Aspergillus* spp. Syntenic analysis of the *A. nidulans AN0273* locus region from AspGD (http://www.aspergillusgenome.org) [71]. (PPTX 158 KB)

Additional file 6: Figure S6: Growth phenotypes of WT and *ΔrsrA* of *A. flavus* and *A. fumigatus*. **(A)** *A. flavus* grown on GMM at 29°C for 7 days. **(B)** Sclerotia formation in *A. flavus* on GMM with 2% sorbitol for 7 days. **(C)** *A. fumigatus* grown on GMM at 37°C for 5 days. (PPTX 696 KB)

Additional file 7: Figure S7: Growth phenotypes of WT and *ΔrsrA* of *A. nidulans, A. fumigatus* and *A. flavus* grown on GMM supplemented with indicated stressors at 37 and 29°C for 2 and 3 days, respectively. (PPTX 162 KB)

Additional file 8: Table S1: Oligonucleotides used in this study. (DOCX 41 KB)
