# Supplementary figures and images for: Illumina identification of RsrA, a conserved C2H2 transcription factor coordinating the NapA mediated oxidative stress signaling pathway in Aspergillus
Source: BMC Genomics. 2014 Nov 22;15(1):1011. doi: 10.1186/1471-2164-15-1011 (PMC4252986; doi:10.1186/1471-2164-15-1011)

## Slide 1
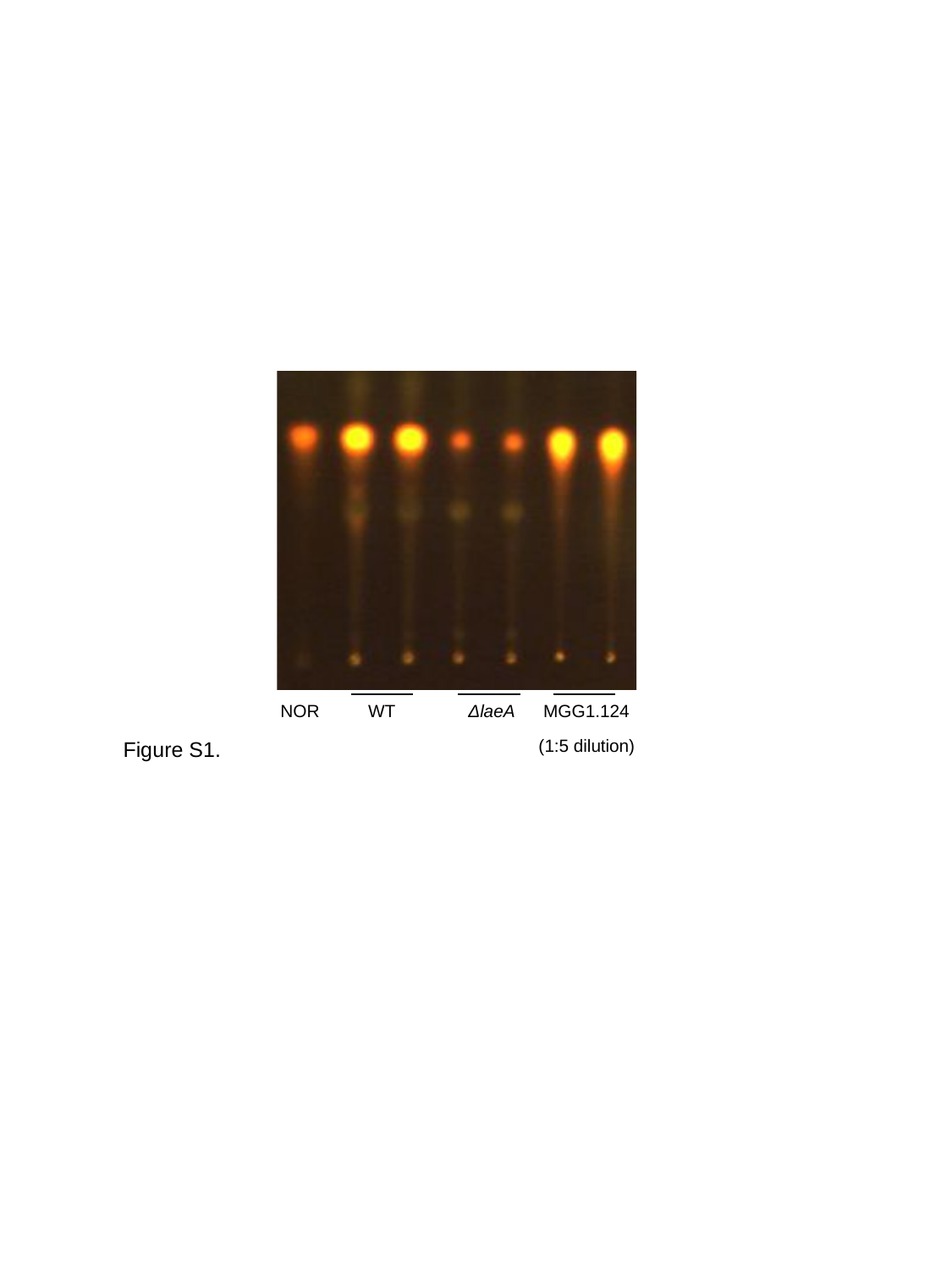

NOR WT ΔlaeA MGG1.124
(1:5 dilution)
Figure S1.

Supplement: Supplementary file 1 — Additional file 1: Figure S1: Thin Layer Chromatography analysis of chloroform extracts for metabolite production by the wild type (WT), ΔlaeA and the 4-NQO generated mutant MGG1.124. Metabolites were extracted from point-inoculated solid cultures grown for 5 days at 37°C in the dark. NOR, Norsolorinic acid. Extracts of MGG1.124 were diluted 1:5 before loading. Extracts from other strains were loaded undiluted. (PPTX 153 KB) [file 12864_2014_6708_MOESM1_ESM.pptx]

## Slide 1
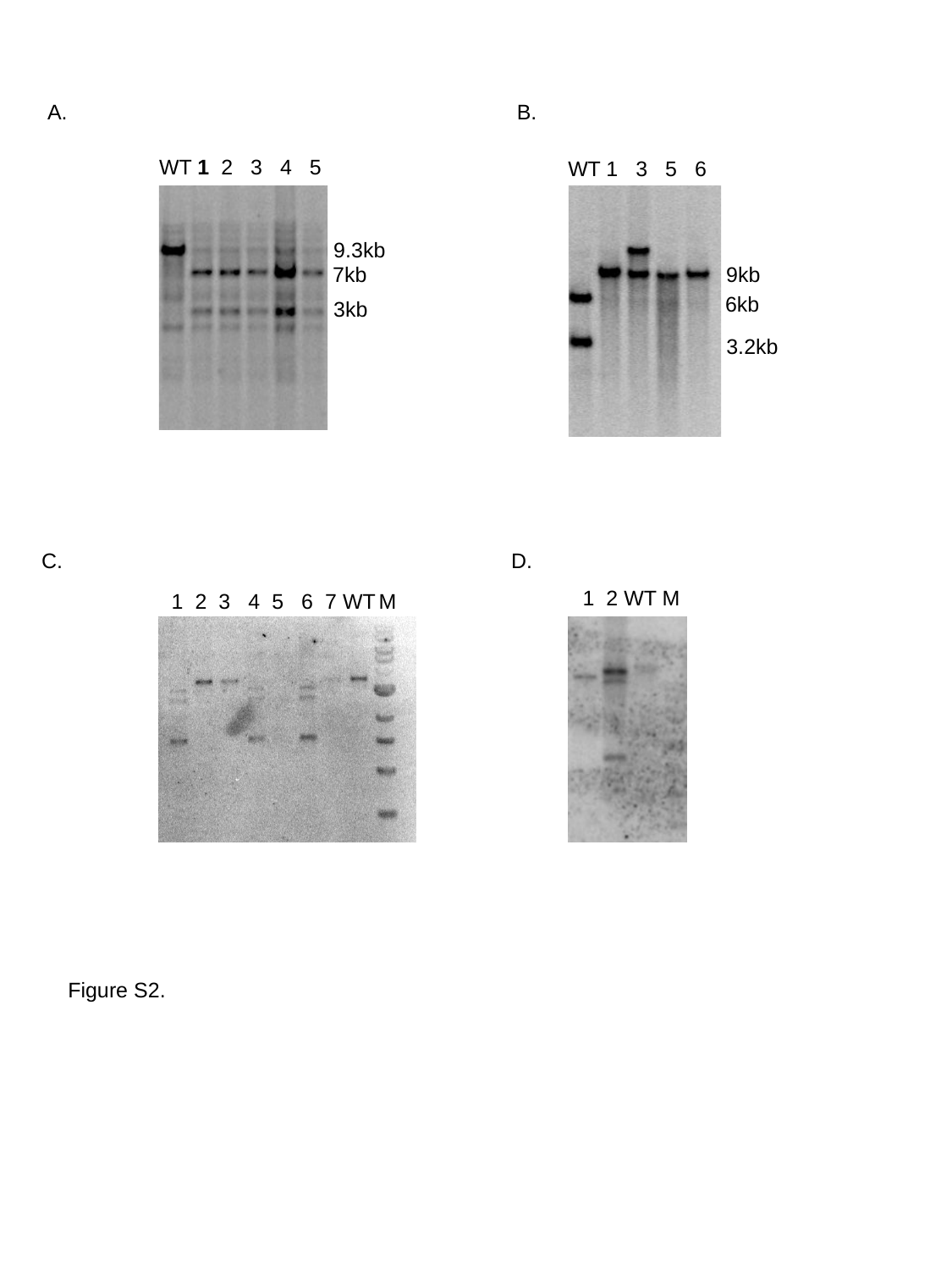

A.
B.
WT 1 2 3 4 5
WT 1 3 5 6
9.3kb
7kb
9kb
6kb
3kb
3.2kb
C.
D.
1 2 WT M
M
1 2 3 4 5 6 7 WT
Figure S2.

Supplement: Supplementary file 2 — Additional file 2: Figure S2: Southern confirmation of rsrA deletion mutant in A. nidulans, A. flavus and A. fumigatus. (A) A. nidulans AN0273: Genomic DNA was digested by NcoI (all correct). #1 for subsequent experiment. WT: 9.3kb, ΔrsrA: 3 and 7kb. (B) A. flavus AFL2G_00759 : Genomic DNA was digested by BglII (#1, 5, 6 correct). #1 for subsequent experiment. WT: 6kb and 3.2kb. ΔrsrA: 9kb (C) A. fumigatus CEA17 AFUB_003250: Genomic DNA was digested with EcoRI (#1, 4, 6 correct). #4 chosen for subsequent experiments. WT: 3.5 kb; ΔrsrA: 2.1, 3.7 and 3.9 kb. (D) A. fumigatus Af293 Afu1g02870: Genomic DNA was digested with HindIII (#2 correct). #2 was chosen for subsequent experiments. WT: 5.5 kb.; ΔrsrA: 1.8, 4.5 and 5 kb. (PPTX 467 KB) [file 12864_2014_6708_MOESM2_ESM.pptx]

## Slide 1
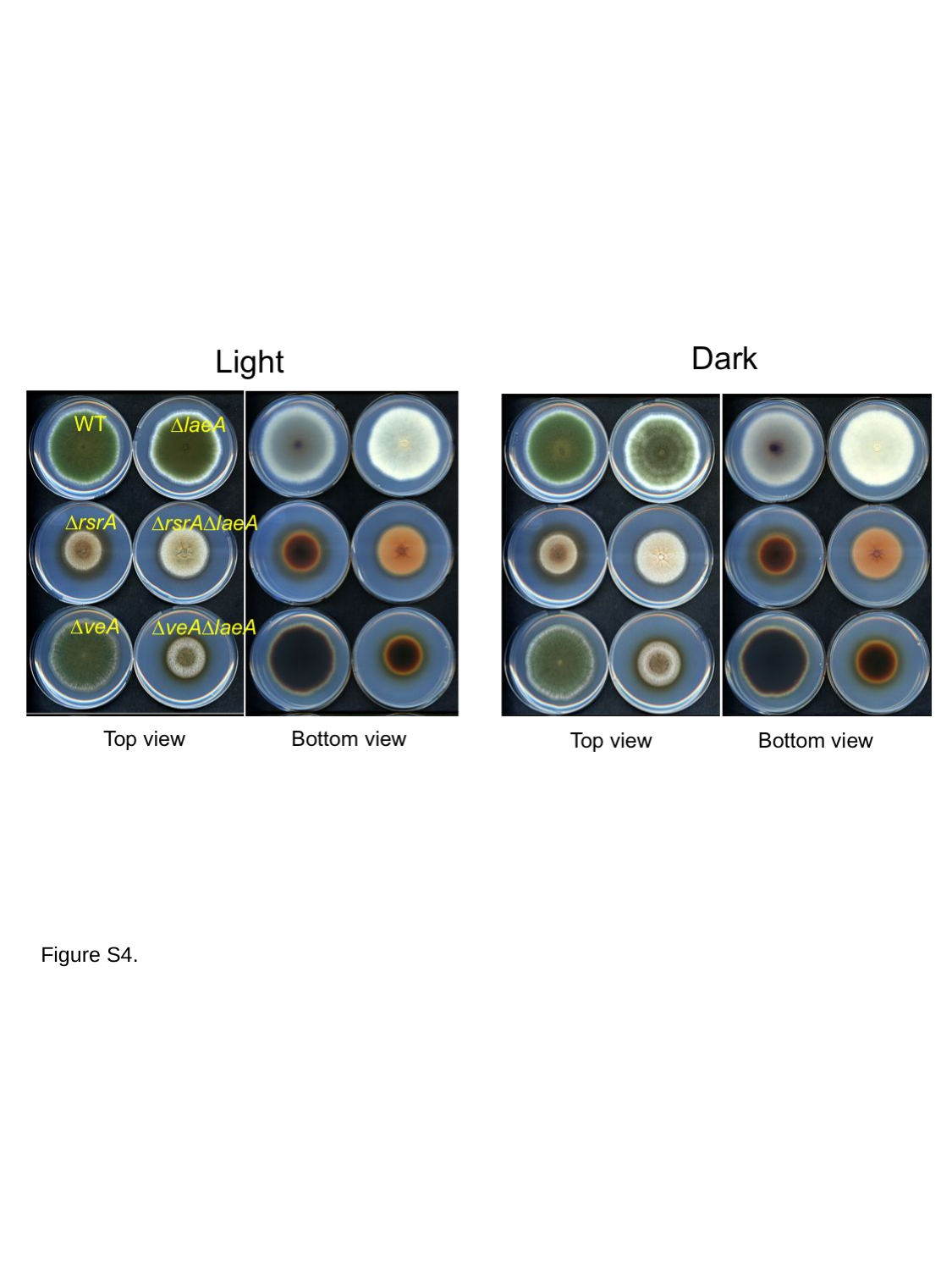

Figure S4.

Supplement: Supplementary file 4 — Additional file 4: Figure S4: Growth phenotypes of WT, ΔlaeA, ΔrsrA, ΔrsrΔlaeA, ΔveA, and ΔveAΔrsrA (RDIT9.32, RJW41A, RJW263.2 and RJW273.17, RJW112.2, and RJW113.4, respectively) strains grown on solid GMM under light and dark at 37°C for 5 days. (PPTX 1 MB) [file 12864_2014_6708_MOESM4_ESM.pptx]

## Slide 1
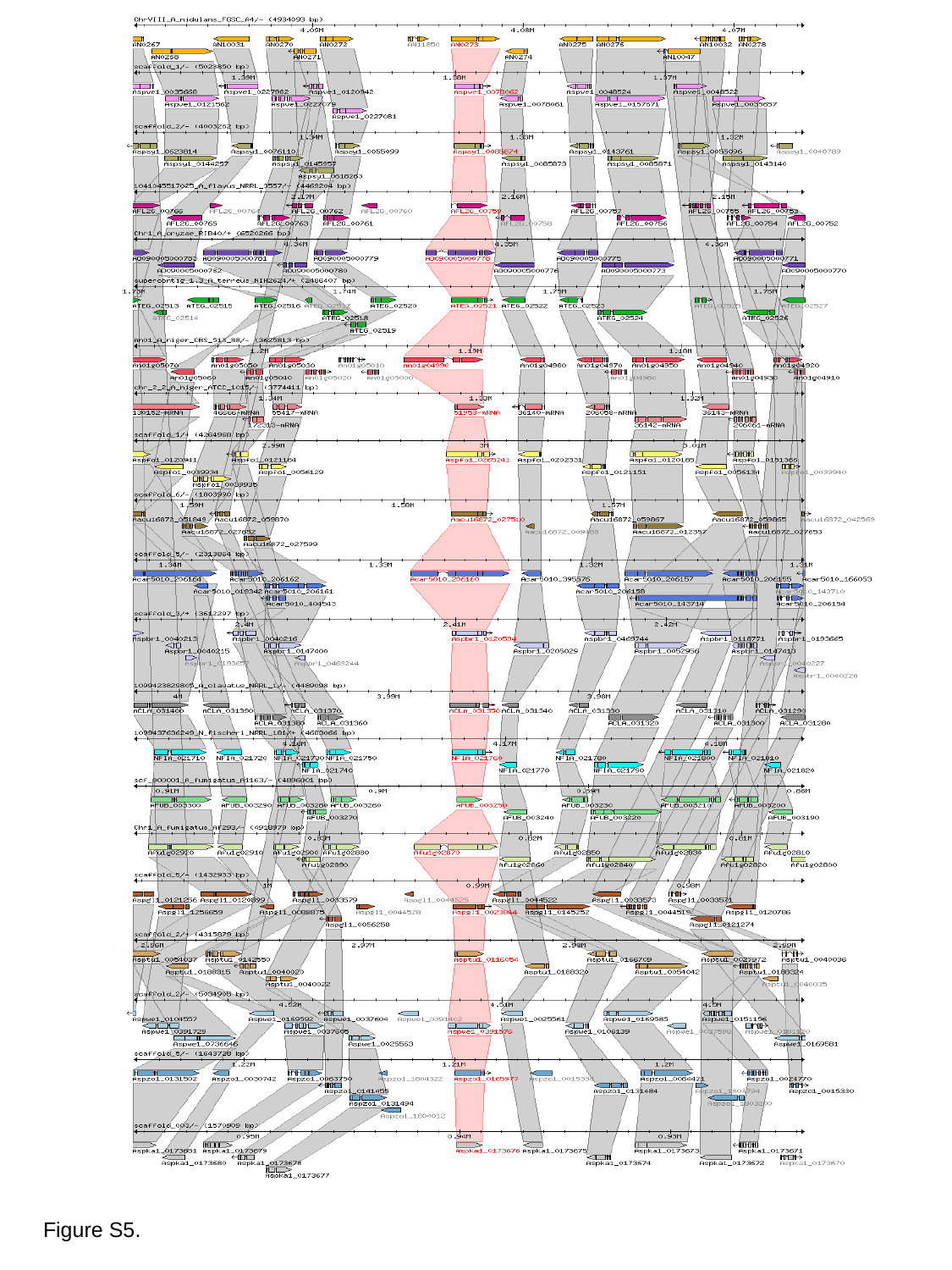

Figure S5.

Supplement: Supplementary file 5 — Additional file 5: Figure S5: Synteny of rsrA in Aspergillus spp. Syntenic analysis of the A. nidulans AN0273 locus region from AspGD (http://www.aspergillusgenome.org) [71]. (PPTX 158 KB) [file 12864_2014_6708_MOESM5_ESM.pptx]

## Slide 1
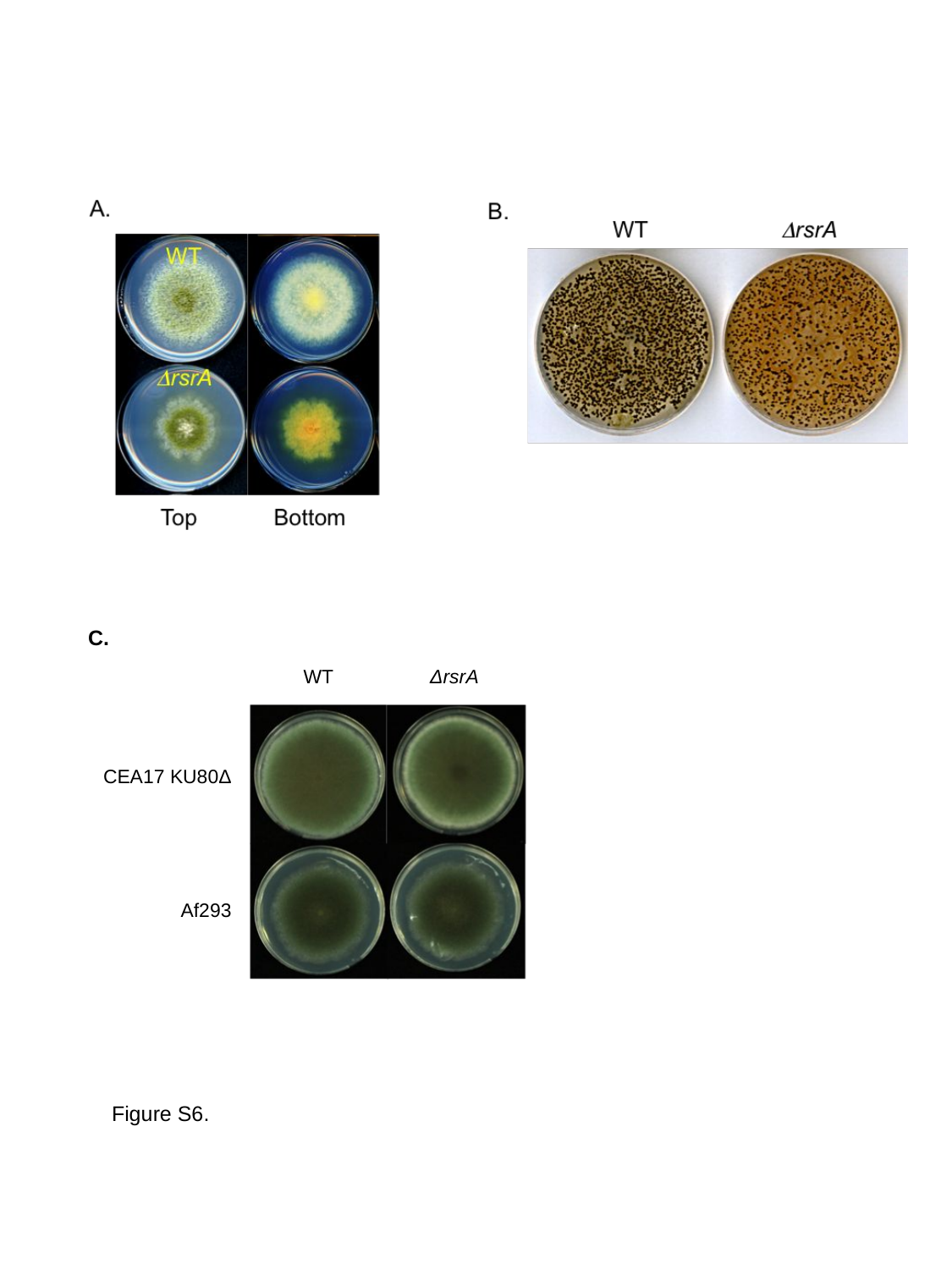

C.
WT
ΔrsrA
CEA17 KU80Δ
Af293
Figure S6.

Supplement: Supplementary file 6 — Additional file 6: Figure S6: Growth phenotypes of WT and ΔrsrA of A. flavus and A. fumigatus. (A) A. flavus grown on GMM at 29°C for 7 days. (B) Sclerotia formation in A. flavus on GMM with 2% sorbitol for 7 days. (C) A. fumigatus grown on GMM at 37°C for 5 days. (PPTX 696 KB) [file 12864_2014_6708_MOESM6_ESM.pptx]
