# Supplementary material for: Illumina identification of RsrA, a conserved C2H2 transcription factor coordinating the NapA mediated oxidative stress signaling pathway in Aspergillus
Source: BMC Genomics. 2014 Nov 22;15(1):1011. doi: 10.1186/1471-2164-15-1011 (PMC4252986; doi:10.1186/1471-2164-15-1011)
Supplement: Supplementary file 3 — Additional file 3: Figure S3: Complementation of ΔrsrA mutant with WT rsrA in A. nidulans. (A) Growth phenotype. (B) Southern confirmation ΔrsrA complementation by restriction enzyme digestion with KpnI and HindIII expected to have 4.7 kb WT rsrA band in ΔrsrA background (3.2 and 2.6 kb bands). (C) TLC analysis of WT, ΔrsrA and complemented strain grown on solid GMM under dark at 37°C for 5 days in triplicate. ST, sterigmatocystin standard. Compl = complemented. (D) Radial growth, (E) Asexual spore production; (F) Sexual spore production. Radial growth and spore counts were measured after 5 days of incubation. Means ± standard deviations are indicated for triplicates of each strain. Levels not connected by same letter are significantly different (P < 0.05) according to Tukey’s multiple comparison test. (PPTX 2 MB) [file 12864_2014_6708_MOESM3_ESM.pptx]

## Slide 1
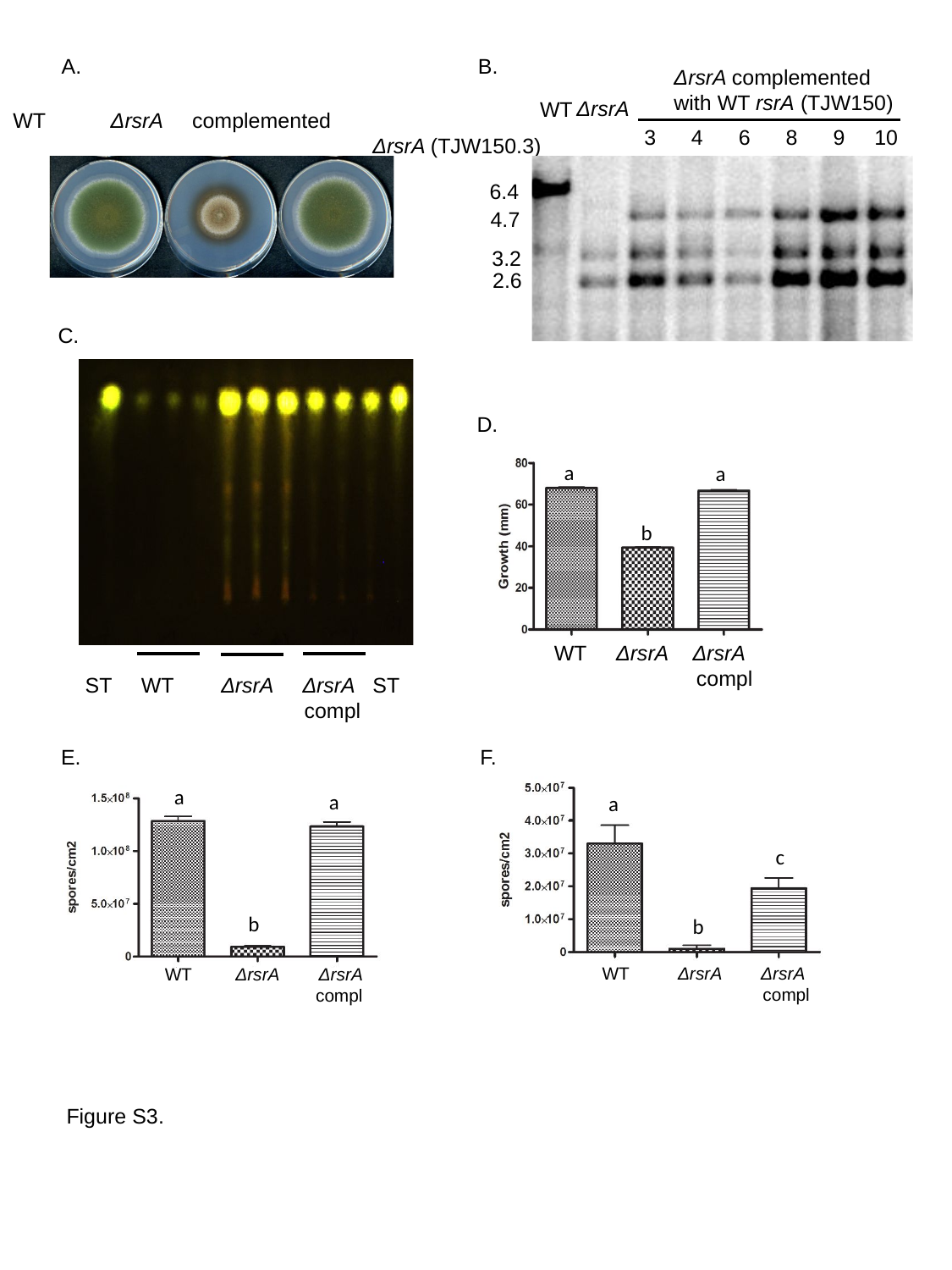

ΔrsrA complemented
with WT rsrA (TJW150)
A.
B.
ΔrsrA
WT
WT ΔrsrA complemented
 ΔrsrA (TJW150.3)
3 4 6 8 9 10
6.4
4.7
3.2
2.6
C.
D.
a
a
b
WT ΔrsrA ΔrsrA
 compl
ST WT ΔrsrA ΔrsrA ST
 compl
F.
E.
a
a
a
c
b
b
WT ΔrsrA ΔrsrA
 compl
WT ΔrsrA ΔrsrA
 compl
Figure S3.
