# Supplementary material for: Illumina identification of RsrA, a conserved C2H2 transcription factor coordinating the NapA mediated oxidative stress signaling pathway in Aspergillus
Source: BMC Genomics. 2014 Nov 22;15(1):1011. doi: 10.1186/1471-2164-15-1011 (PMC4252986; doi:10.1186/1471-2164-15-1011)
Supplement: Supplementary file 7 — Additional file 7: Figure S7: Growth phenotypes of WT and ΔrsrA of A. nidulans, A. fumigatus and A. flavus grown on GMM supplemented with indicated stressors at 37 and 29°C for 2 and 3 days, respectively. (PPTX 162 KB) [file 12864_2014_6708_MOESM7_ESM.pptx]

## Slide 1
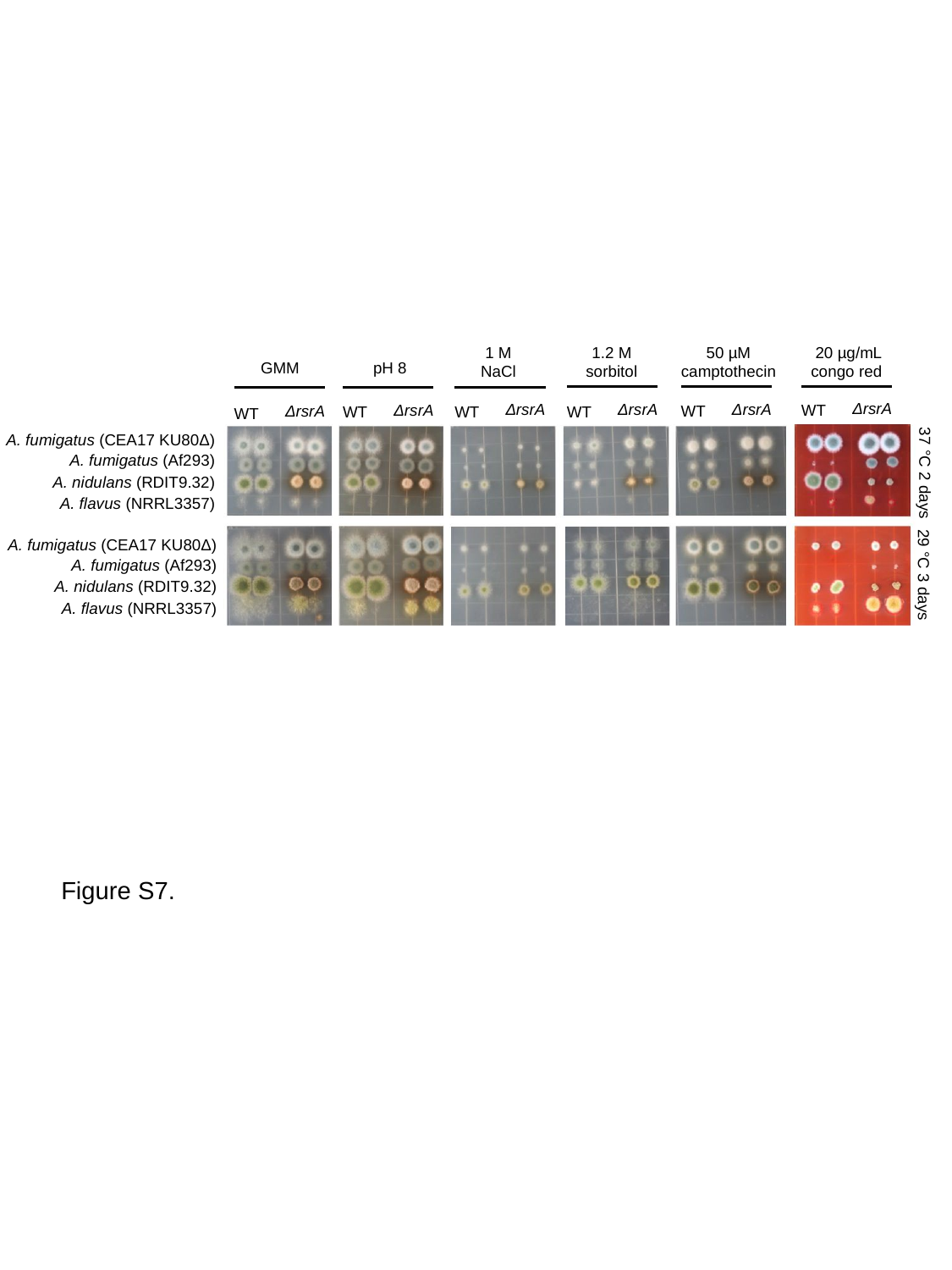

1 M
NaCl
 20 µg/mL
congo red
1.2 M
sorbitol
50 µM
camptothecin
pH 8
GMM
ΔrsrA
WT
ΔrsrA
WT
ΔrsrA
WT
ΔrsrA
WT
ΔrsrA
WT
ΔrsrA
WT
A. fumigatus (CEA17 KU80Δ)
A. fumigatus (Af293)
A. nidulans (RDIT9.32)
A. flavus (NRRL3357)
37 °C 2 days
A. fumigatus (CEA17 KU80Δ)
A. fumigatus (Af293)
A. nidulans (RDIT9.32)
29 °C 3 days
A. flavus (NRRL3357)
Figure S7.
