# Supplementary material for: Illumina identification of RsrA, a conserved C2H2 transcription factor coordinating the NapA mediated oxidative stress signaling pathway in Aspergillus
Source: BMC Genomics. 2014 Nov 22;15(1):1011. doi: 10.1186/1471-2164-15-1011 (PMC4252986; doi:10.1186/1471-2164-15-1011)
Supplement: Supplementary file 8 — Additional file 8: Table S1: Oligonucleotides used in this study. (DOCX 41 KB) [file 12864_2014_6708_MOESM8_ESM.docx]

Supplementary Table

Table S1 Oligonucleotides used in this study.

| Name | Sequence | Use |
| --- | --- | --- |
| *A. nidulans rsrA* deletion | | |
| 0273F5 | TGACTAGACTACGTAGCCGTCG | gene deletion |
| 0273PR5 | CGATATCAAGCTATCGATACCTCGACTCTCAGTCATCATGGAGGCGGTTTTTGGCG | gene deletion |
| pyrGF | GAGTCGAGGTATCGATAGCTTG | gene deletion |
| pyrGR | ATTCGACAATCGGAGAGGCTGC | gene deletion |
| 0273PF3 | GTCGCTGCAGCCTCTCCGATTGTCGAATCATCCACCCTTAAATGATGCCTGCTCC | gene deletion |
| 0273R3 | AAGGACCGTGATCGTCATGAGG | gene deletion |
| 0273IF | TTGGACTCGAGGAGGTCAAACCC | rsrA probe |
| 0273IR | AATTGTGAACGAGCCCATGCCG | rsrA probe |
| *A. nidulans rsrA* complementation | | |
| HindrrpAcompF | ACGGTCGGTCaagctTACTGTGTTAGTCCTGTGCTAGTCGC | complementation |
| HindrrpAcompR | CCGCGGATTCAagctTAAGAAGGACCGTGATCGTCATGAGGC | complementation |
| kpnrrpAcompF | ACGGTCGGTCggtaccACTGTGTTAGTCCTGTGCTAGTCGC | complementation |
| kpnrrpAcompR | CCGCGGATTCggtaccAAGAAGGACCGTGATCGTCATGAGGC | complementation |
| HindrsrAcompF | ACGGTCGGTCaagctTACTGTGTTAGTCCTGTGCTAGTCGC | complementation |
| KpnrsrAcompR | CCGCGGATTCggtaccAAGAAGGACCGTGATCGTCATGAGGC | complementation |
| *A. flavus rsrA* deletion | | |
| fgaeA5F | ACATCGCATTGCTGGTGTCTCGC | gene deletion |
| fgaeA5R | CAAGCTATCGATACCTCGACTCTAAAGATGCAGAATAAGCTCACCTGCTGAAGG | gene deletion |
| pyrGF | GAGTCGAGGTATCGATAGCTTG | gene deletion |
| pyrGR | ATTCGACAATCGGAGAGGCTGC | gene deletion |
| fgaeA3F | GCAGCCTCTCCGATTGTCGAATTCTCAGCACTTCGCCATCATATATGACTCTCG | gene deletion |
| fgaeA3R | TCTTCTGATCGTTGCTTATGTTGGG | gene deletion |
| fgaeAorfF | TCATATGATCCCGACGAGGACC | probe |
| fgaeAorfR | AGTTTGGTCTGTGGGTGTTGGG | probe |
| *A. fumigatus rsrA* deletion | | |
| Afu1g02870KO5F | GTCGTGGTTATCATCCTCATC | gene deletion |
| Afu1g02870KO5R | CCAATTCGCCCTATAGTGAGTCGTATTACGGAGCGGGTAGCTAATTCAGC | gene deletion |
| Afu1g02870KO3F | GCTGTCGCTGCAGCCTCTCCGATTGTCGAATGCCAAGATGACCCCCGTTG | gene deletion |
| Afu1g02870KO3R | GTATGTATGCATTTATGGCGGG | gene deletion |
| PWpyrGpromF | CGTAATACGACTCACTATAGGG | gene deletion |
| PWpyrGtermR | ATTCGACAATCGGAGAGGCTGC | gene deletion |
| SNP candidate confirmation | | |
| 6248F | TTTCTGGCATTGTTCGTCGGC | AN6248 PCR |
| 6248R | CTCAATTAGTGCCATCCGTGC | AN6248 PCR |
| 6208F | TGCATAAGAGCCACCTCCTCG | AN6208 PCR |
| 6208R | ATATTCCAGGAGTGCCATGGG | AN6208 PCR |
| 6205F | TTTTCAGGTTCGCTTTTCTCCC | AN6205 PCR |
| 6205R | GAGGTCGTTAGGTAGTGTCGG | AN6205 PCR |
| 3763F | TGAACCAATGACATGGTGGGC | AN3763 PCR |
| 3763R | ACGATCTGGATTCGTCACCC | AN3763 PCR |
| 5330F | TCGAGGATACAGTACCCTTGG | AN5330 PCR |
| 5330R | ACCACTTATACCGGTCTGGC | AN5330 PCR |
| 5318F | AATCTTACCGGTCTCTGACCG | AN5318 PCR |
| 5318R | TGGCAGTTTGTCTTTCCTTGCC | AN5318 PCR |
| 5313F | ACTTGCTTCCTCTCCGATAGC | AN5313 PCR |
| 5313R | AATAGAGTGCAACAGGCTGCC | AN5313 PCR |
| 1594F | TCATGTGCGAGATCACATCCG | AN1594 PCR |
| 1594R | AGAACGAGCTTCAGGTCTTCC | AN1594 PCR |
| 0324F | TCGAAGTTGGTTGTAGAGCGC | AN0324 PCR |
| 0324R | TCGTACCTGGTGTGTATTGGG | AN0324 PCR |
| 0273F | ATTGACTAGGGCTGACAACGG | AN0273 PCR |
| 0273R | CCACCCATAGATAACACTCGG | AN0273 PCR |
| Anid napA int FOR | GGCGAACTACCTGGGAGTGC | Anid napA PCR |
| Anid napA int REV | GAATGCTCTCTGCGCAGCACG | Anid napA PCR |
| Anid atfA int FOR | GCCGTGGCTTCGGCAGTCTC | Anid atfA PCR |
| Anid atfA int REV | GTGGCAGCCGATGGGTTAGG | Anid atfA PCR |
| Anid nkuA int FOR | GAGGAGGAGCTGGATGAGGC | Anid nkuA PCR |
| Anid nkuA int REV | CATGTCCCGGGGGTTGGAG | Anid nkuA PCR |
| Northern probes | | |
| veAF | ATACCTGGATAAACCAAATCGAGC | veA probe |
| veAR | AGGTTCATTCGCAGGGCTAGAC | veA probe |
| laeAF | ATACCTGGATAAACCAAATCGAGC | laeA probe |
| laeAR | AGGTTCATTCGCAGGGCTAGAC | laeA probe |
| hapCF | AGGAGTAGTGTTTCTCGTGATGC | hapC probe |
| hapCR | ATAAGATTCGCCACCAGCTCCG | hapC probe |
| napAF | GGCGAACTACCTGGGAGTGC | napA probe |
| napaR | GAATGCTCTCTGCGCAGCACG | napA probe |
| trxAF | ACATAGTCTTCGTCCGTCAGCC | trxA probe |
| trxAR | GTTTCATACAGACCAGAGTCTGC | trxA probe |
| trxRF | TCAAGCCTGTCCTTTACGAGGG | trxR probe |
| trxRR | AGCAGCAATGCAACCAGATCCC | trxR probe |
| prxAF | ATGTCTGGACTTAAGGCCGGTG | prxA probe |
| prxAR | CAGGTGCTTGATGACAGTCTCG | prxA probe |
| gpxAF | TCAGATCTAGTTCCTCTTCGACC | gpxA probe |
| gpxAR | ATAGCCTTCGAAGCCAGAAGCC | gpxA probe |
| catBF | TACCTTGACACCCAGCTCAACC | catB probe |
| catBR | TCTATTCATCCGAGTCCAGGGC | catB probe |
| glrAF | TGGAACTTCGCTTCCATCACCG | glrA probe |
| glrAR | ACCTGTGACATCGCCAATAGCG | glrA probe |
| gpdAF | GCTAACGTCGTGTGATGTAGGC | gpdA probe |
| gpdAR | CTTGAGCTCGTTCTCAGAAGCC | gpdA probe |
| sodAF | TAAATCATTGGATGCGGTAGCGG | sodA probe |
| sodAR | CGAGATGAAGAATGCTCACCGC | sodA probe |
| gstAF | AGCAAGAACGTCCAGAAGGAGC | gstA probe |
| gstAR | ATTCTTCATCCCCTCCTGCACC | gstA probe |
